# Supplementary material for: Deep learning reduces sensor requirements for gust rejection on a small uncrewed aerial vehicle morphing wing
Source: Commun Eng. 2024 Mar 21;3:53. doi: 10.1038/s44172-024-00201-8 (PMC10957986; doi:10.1038/s44172-024-00201-8)
Supplement: Supplementary file 2 — Supplementary Information [file 44172_2024_201_MOESM2_ESM.pdf]

# Supplementary Information for

## Deep learning reduces sensor requirements for gust rejection on a small uncrewed aerial vehicle morphing wing

Kevin P.T. Haughn
Christina Harvey
Daniel J. Inman

Corresponding author: kevin.p.haughn.civ@army.mil

**The PDF file includes:**

Supplementary Figs. 1 to 19
Supplementary Table 1 and 2

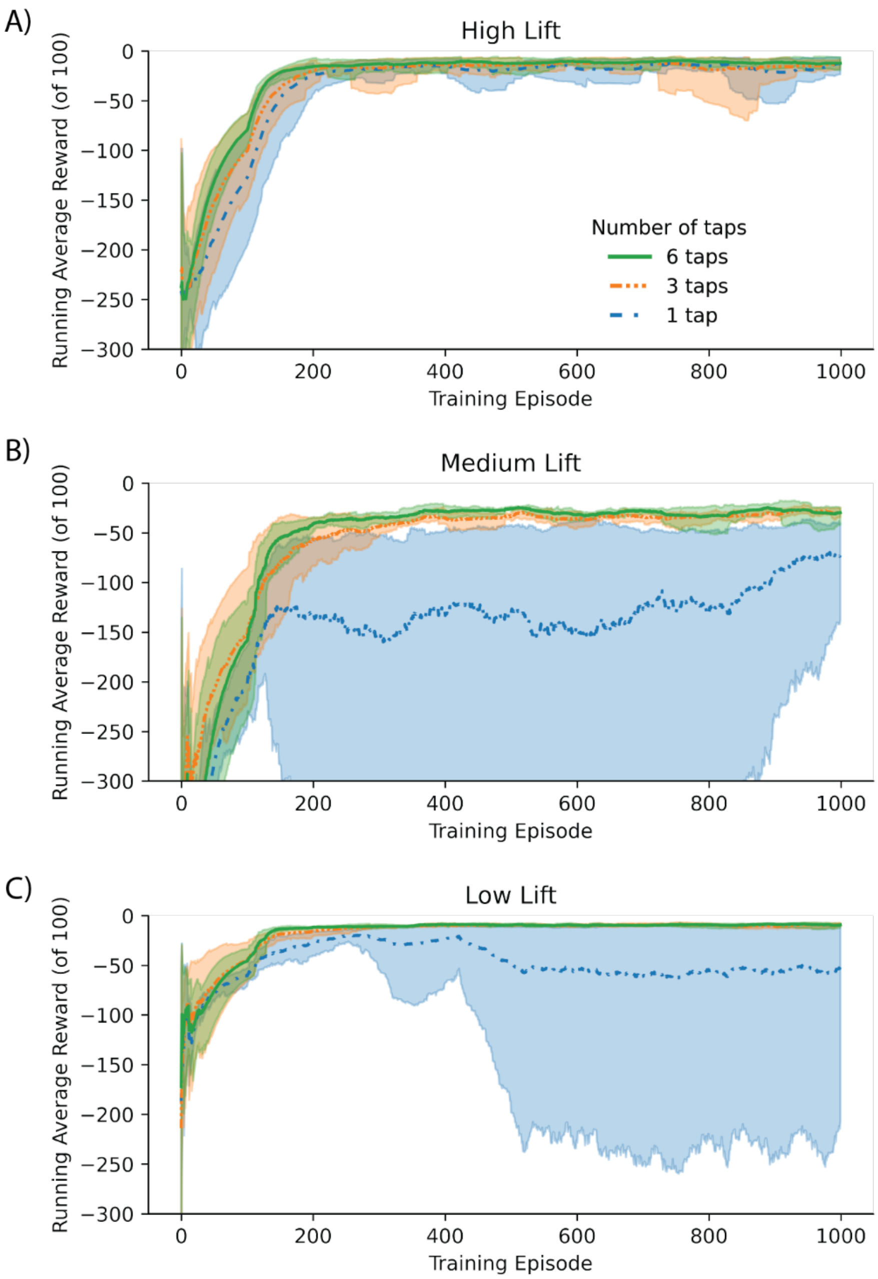


**Supplementary Fig. 1.**

Running average reward earned during proximal policy optimization (PPO) training when using six (green), three (orange), and one (blue) pressure tap(s) for the (**A**) high-lift, (**B**) medium-lift, and (**C**) low-lift flight conditions. Shaded area represents maximum and minimum (high lift: n=10, medium lift: n=5, low lift: n=5) reward values for respective training episodes during training.

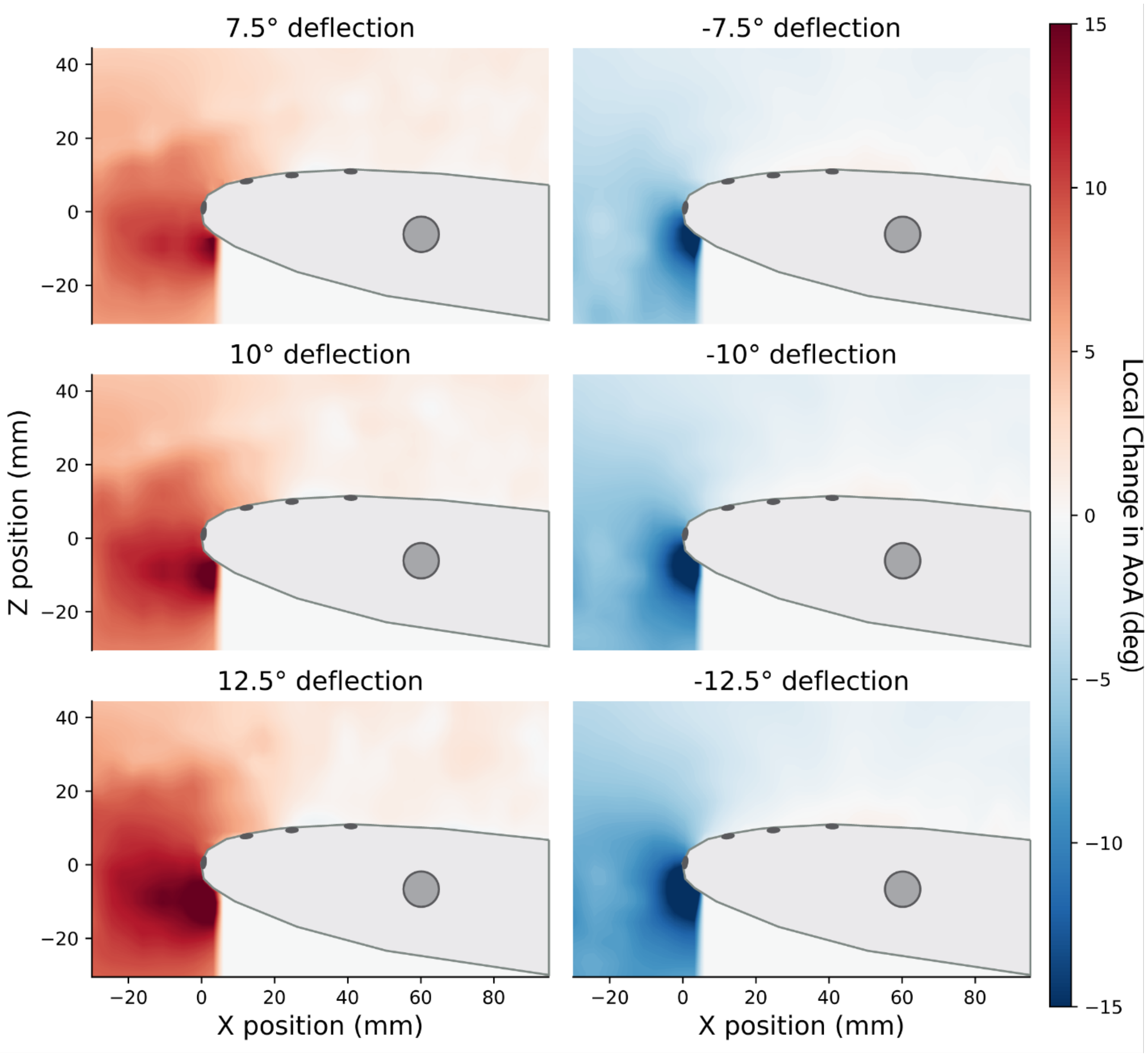


**Supplementary Fig. 2.**

The local angles of attack (AoA) perturbations of the incoming flow as generated by the various gust deflections were measured using particle image velocimetry (PIV) to find the difference between local AoA values during baseline and gusting conditions. Positive gust generator deflections produced increasingly positive changes in local AoA, and negative gust generator deflections produced increasingly negative changes in local AoA. This is representative of what would be experienced as an updraft or downdraft in nature.

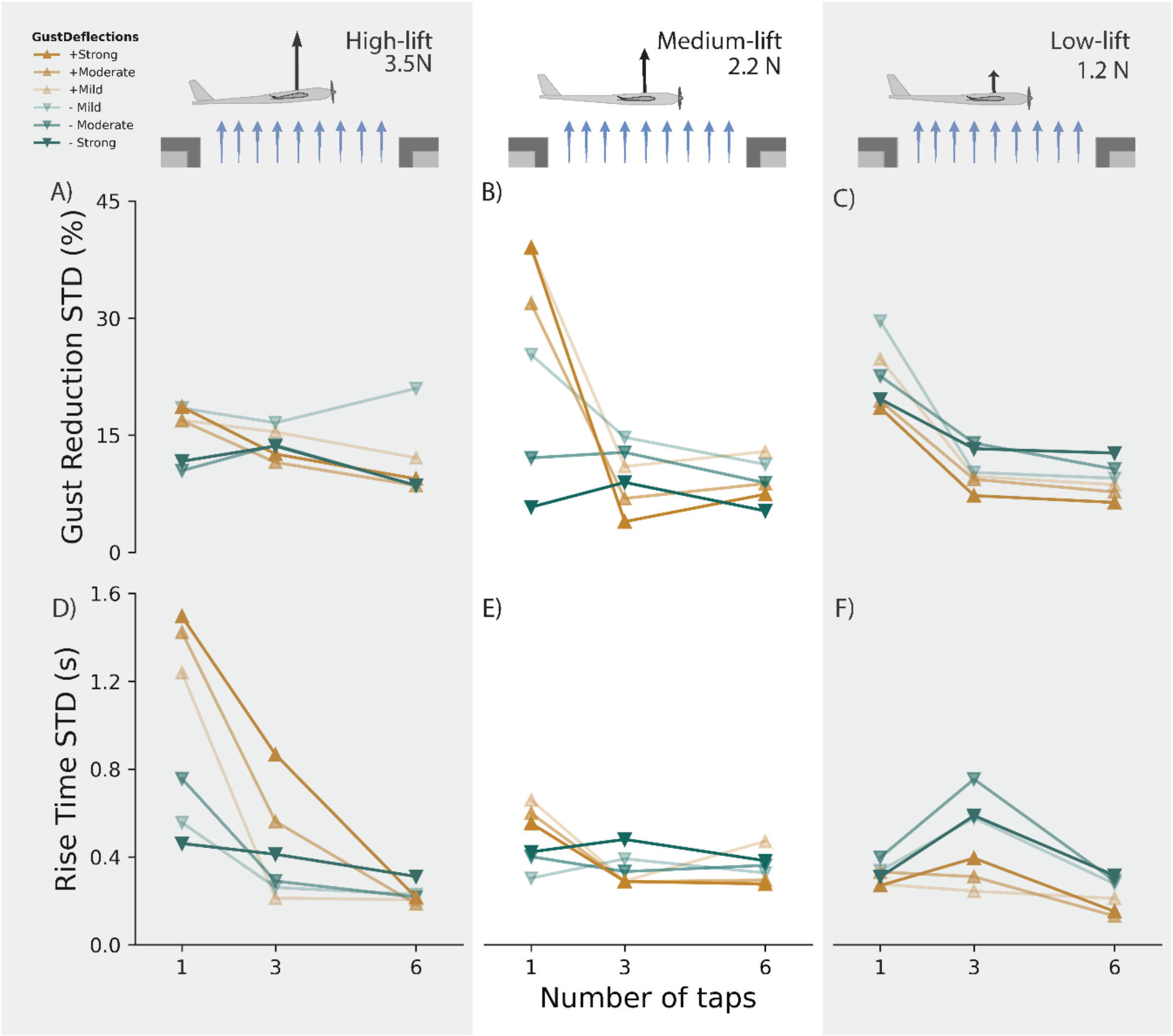


**Supplementary Fig. 3.**

Standard deviations of (**A**,**B**,**C**) settled gust rejection percentage (GRP) and (**D**,**E**,**F**) rise time represent overall performance consistency for controllers with each tested number of pressure taps. Values shown are the standard deviations for all trained controllers for each gust condition and flight configuration.

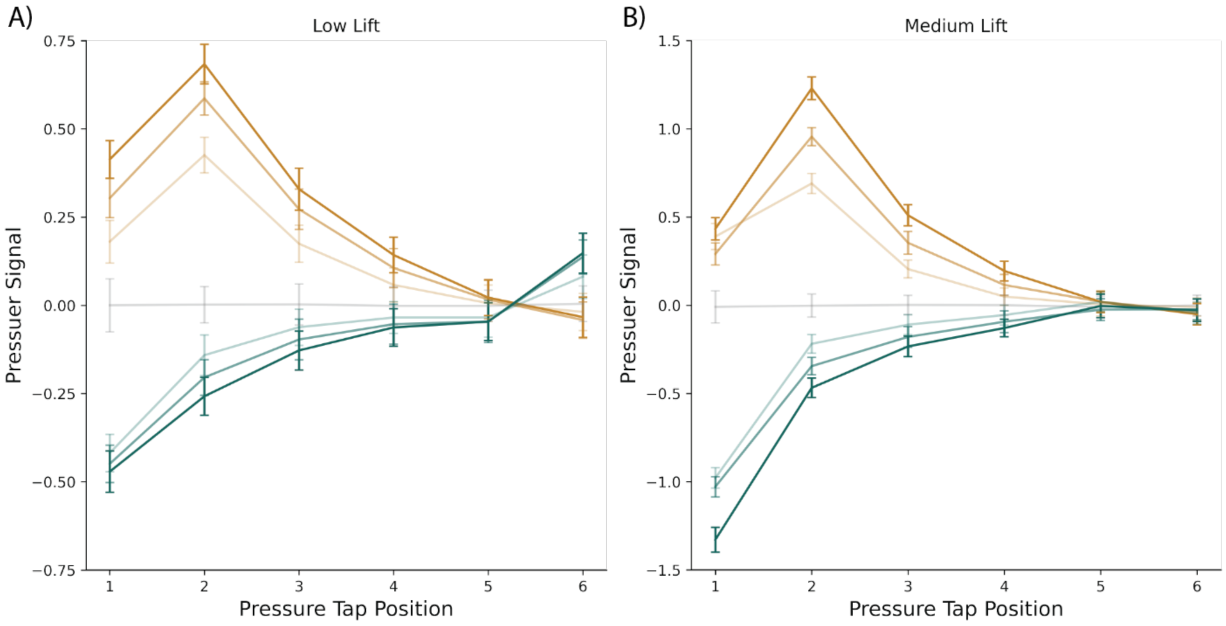


**Supplementary Fig. 4.**

Pressure taps for the (**A**) low lift and (**B**) medium lift flight conditions show similar sensitivity degradation for rearward pressure tap locations (4,5,6). Error bars represent 95% confidence intervals.

A) 6-tap STD: 3.9%

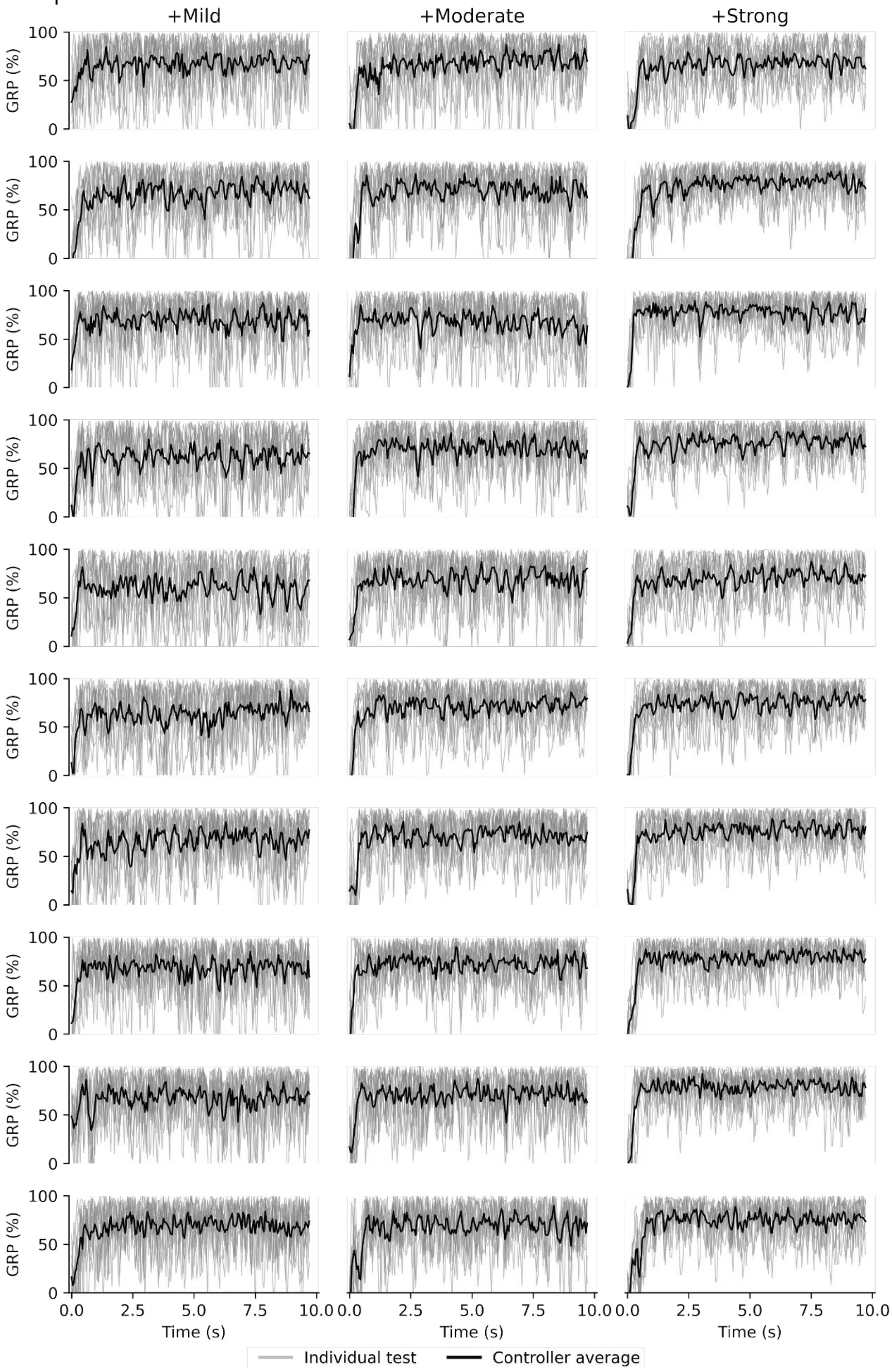

+Mild
+Moderate
+Strong
GRP (%)
100
50
0
0.0
2.5
5.0
7.5
10.0
Time (s)
Individual test
Controller average

B)

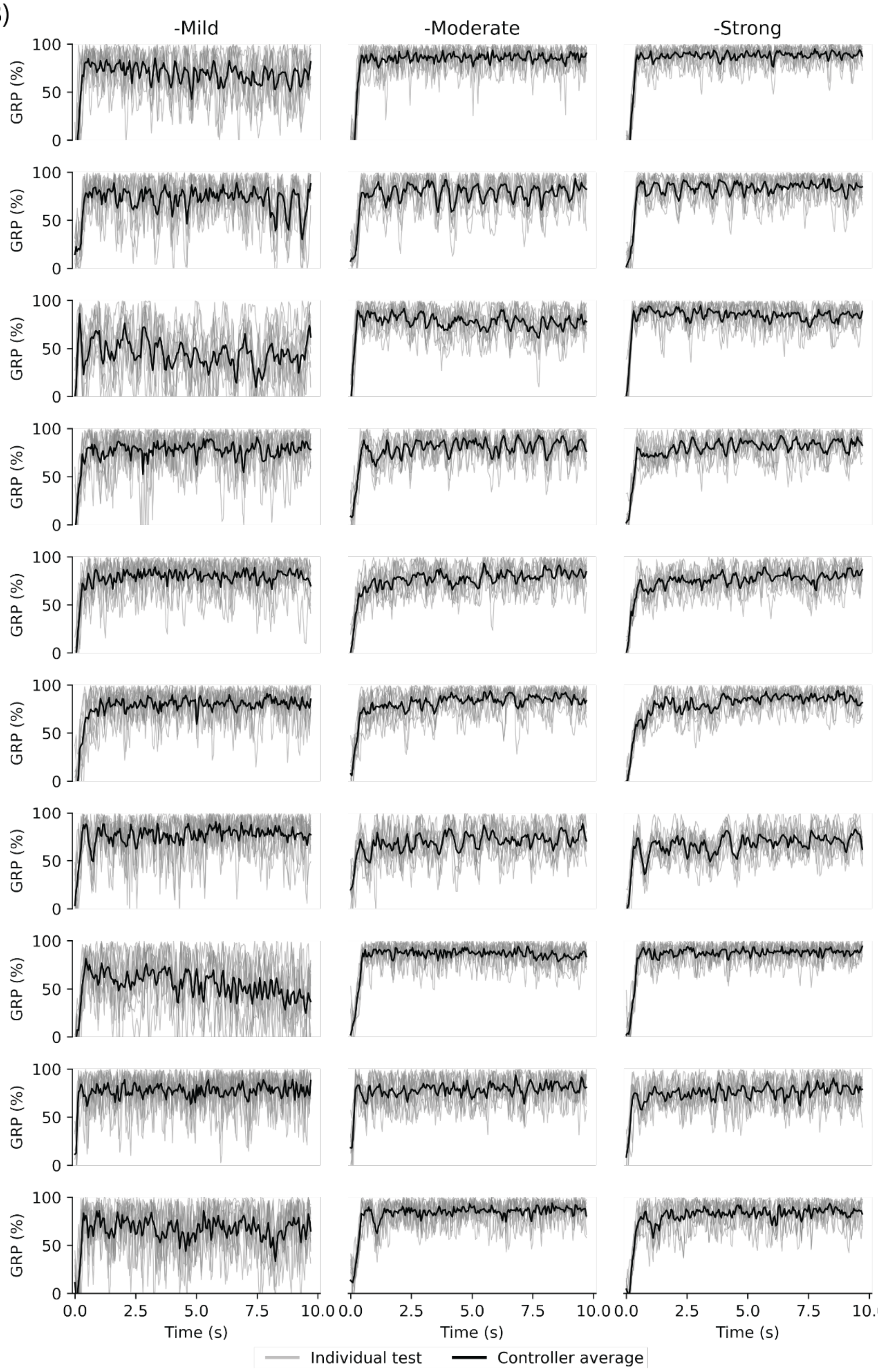
-Mild
-Moderate
-Strong
GRP (%)
Time (s)
Individual test
Controller average

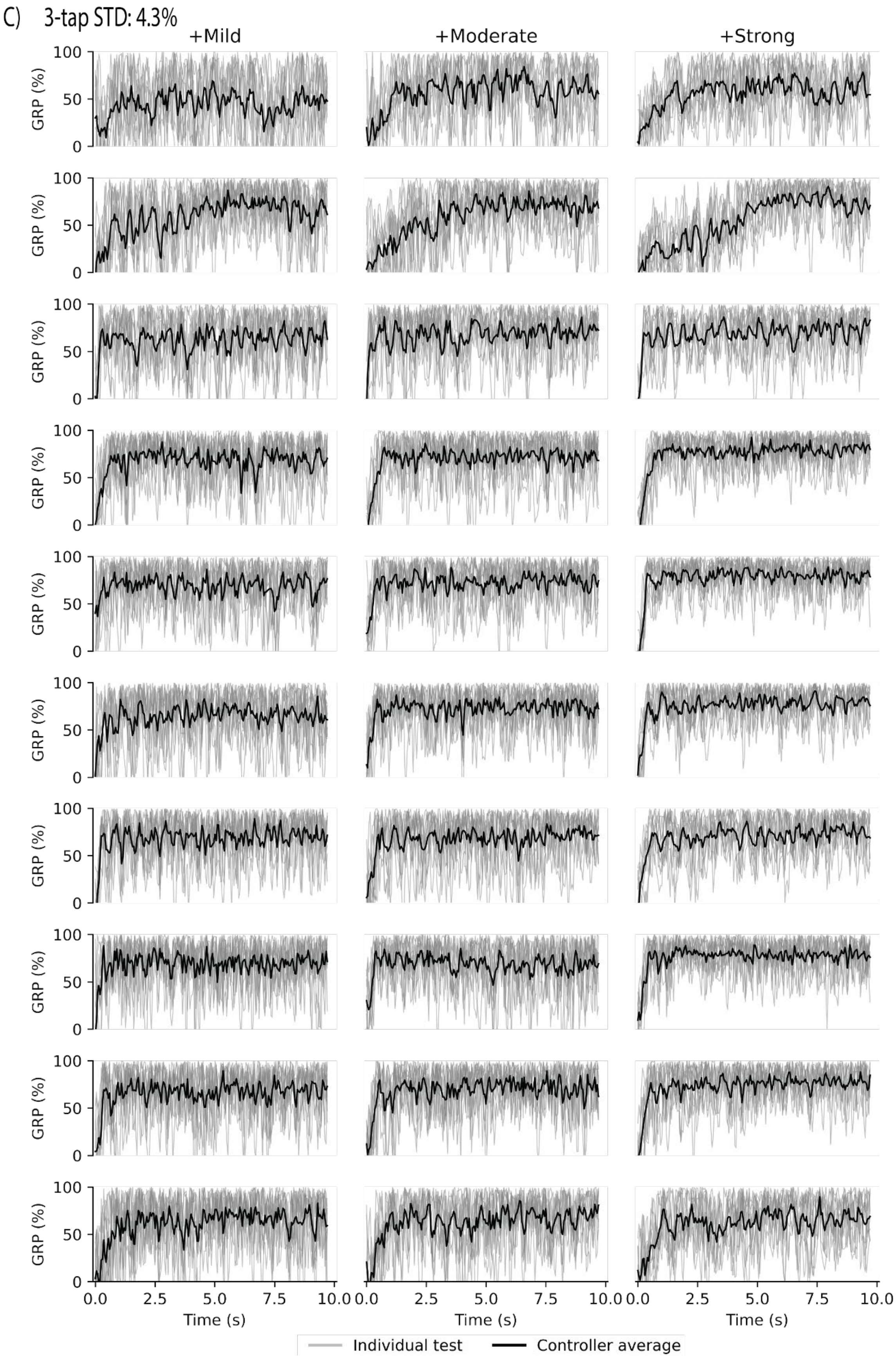
C) 3-tap STD: 4.3%
+Mild
+Moderate
+Strong
GRP (%)
Time (s)
Individual test
Controller average

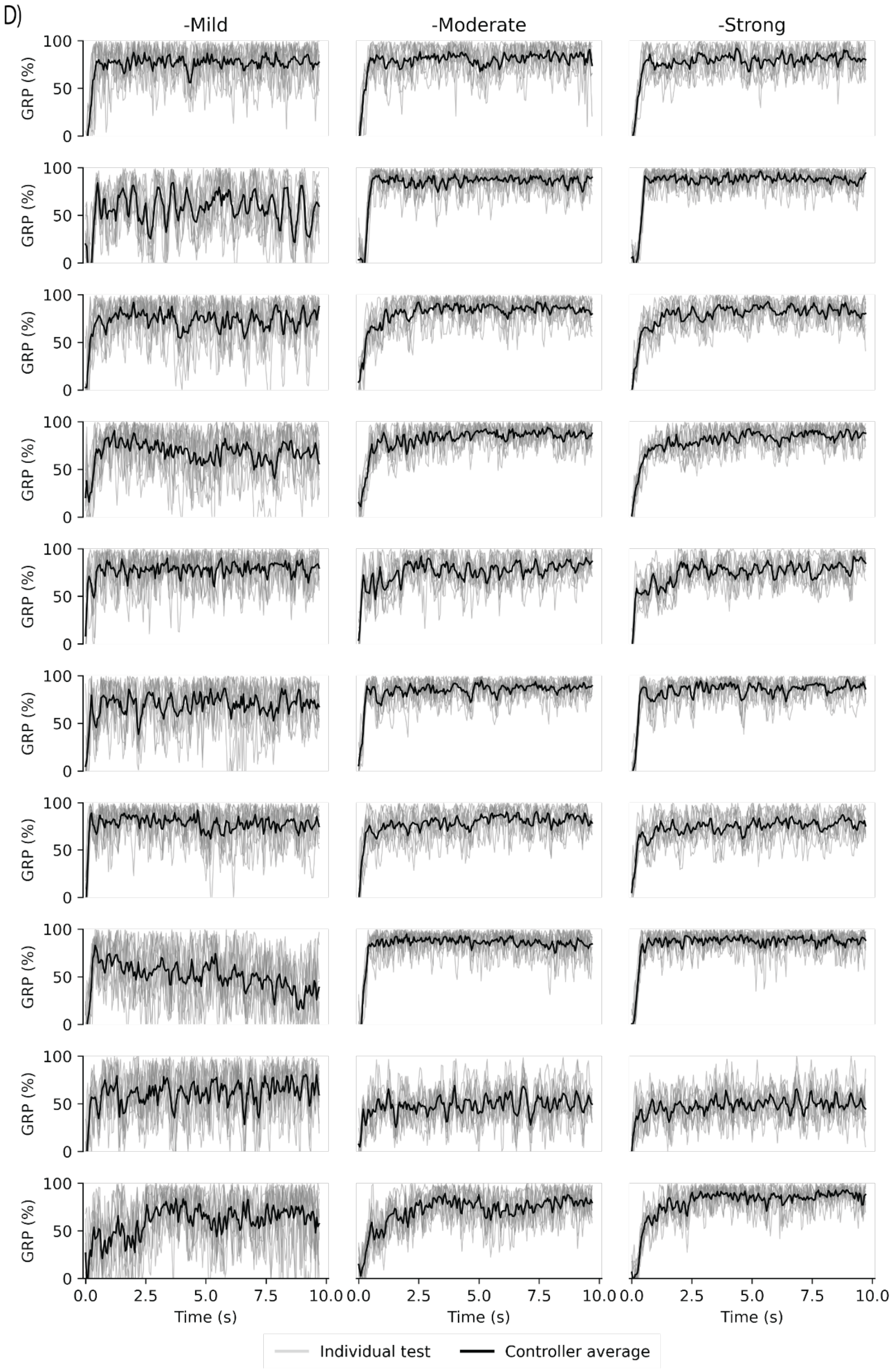
D)
-Mild
-Moderate
-Strong
GRP (%)
100
50
0
0.0
2.5
5.0
7.5
10.0
Time (s)
Individual test
Controller average

E) 1-tap STD: 4.8%

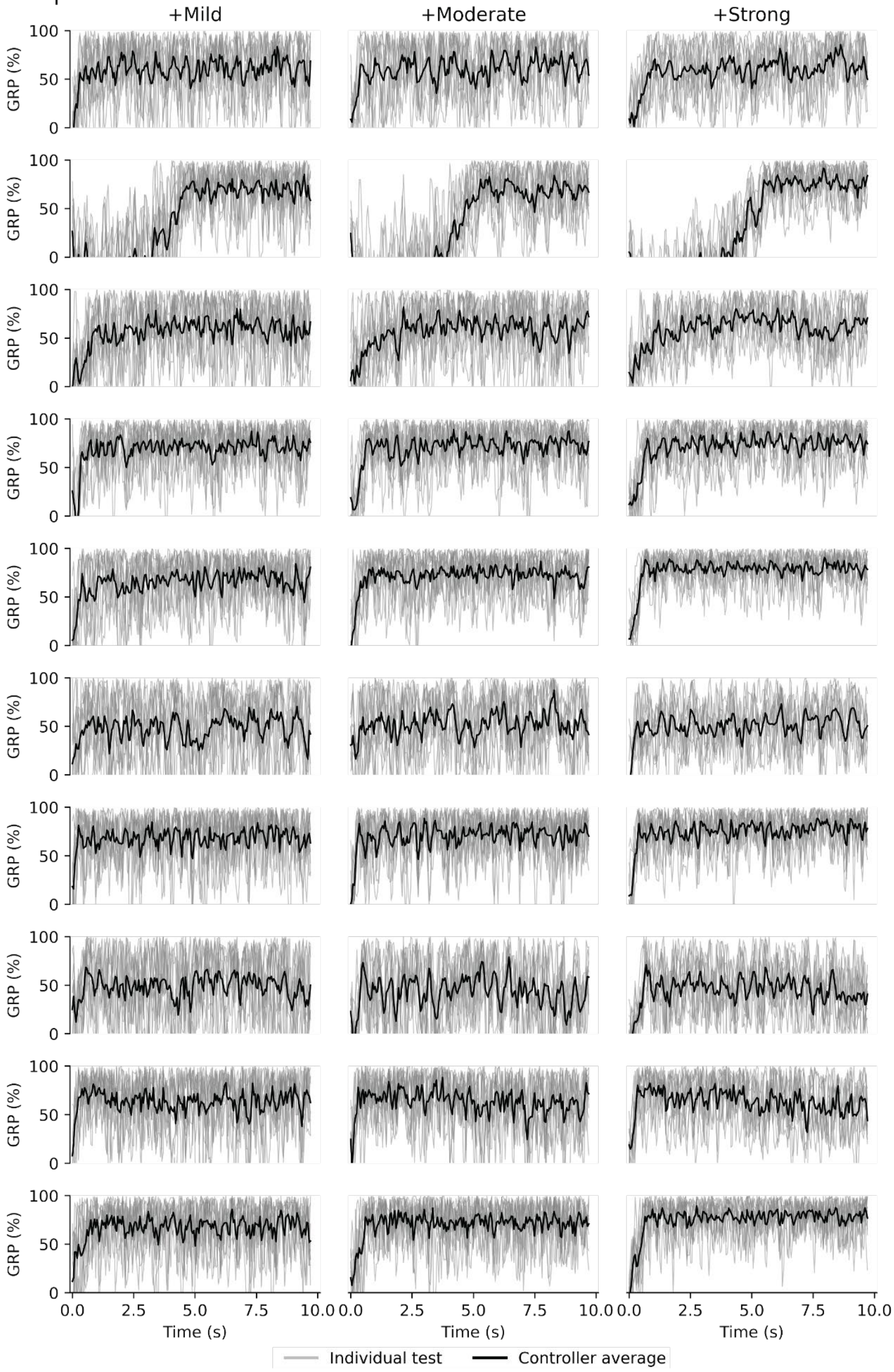
+Mild
+Moderate
+Strong
GRP (%)
Time (s)
Individual test
Controller average

F)

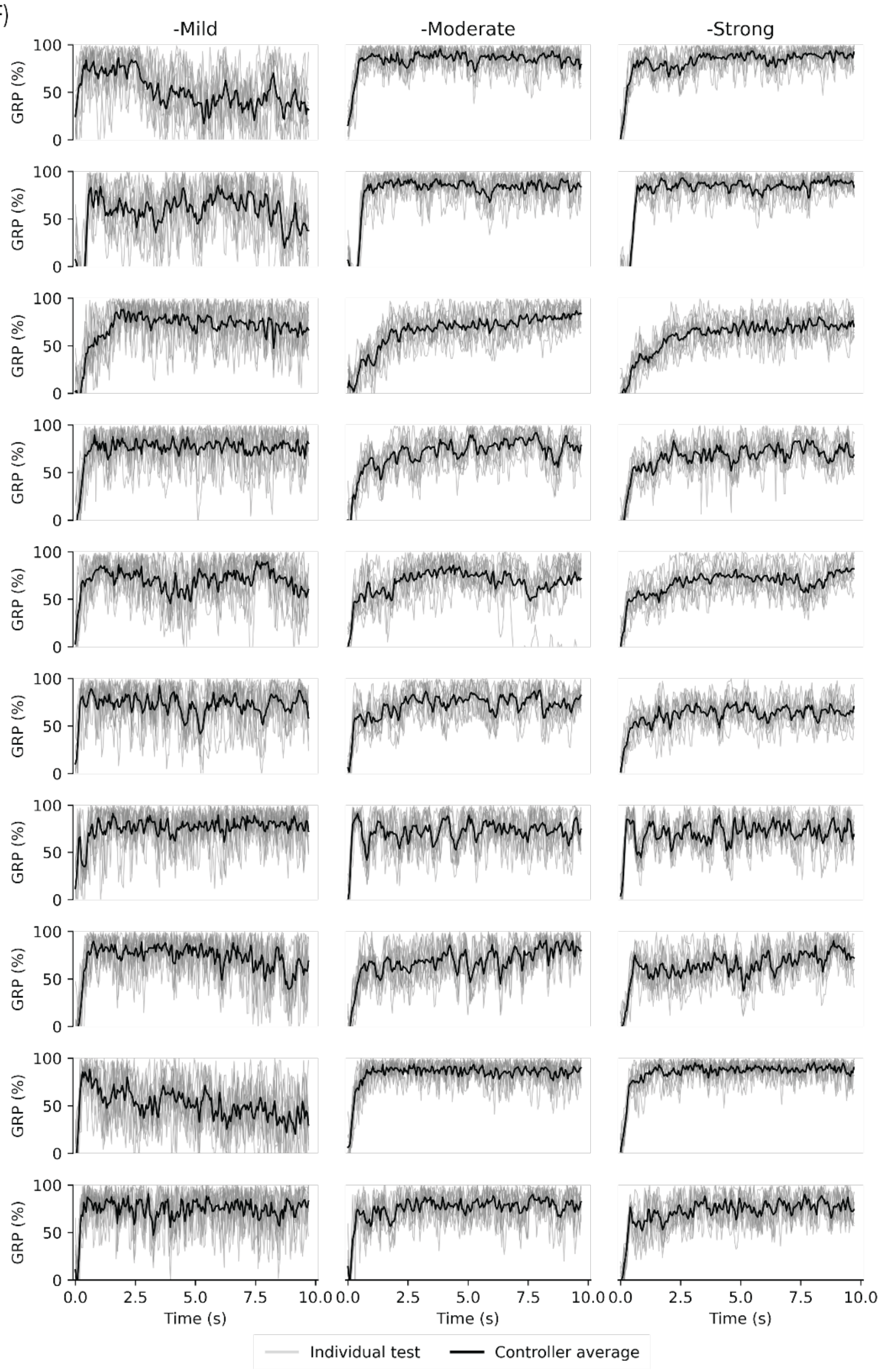


**Supplementary Fig. 5.**

GRP of each test iteration (10), during each gust condition (6), for each trained controller (10), using (**A**, **B**) six, (**C**, **D**) three, (**E**, **F**) and one pressure tap(s) for the high-lift flight configuration. Individual tests (gray) were averaged (black) to provide a general gust rejection percentage (GRP) for each trained controller. The average standard deviation (STD) represents performance consistency of individual trained controllers between individual tests for each pressure tap configuration at each gust condition.

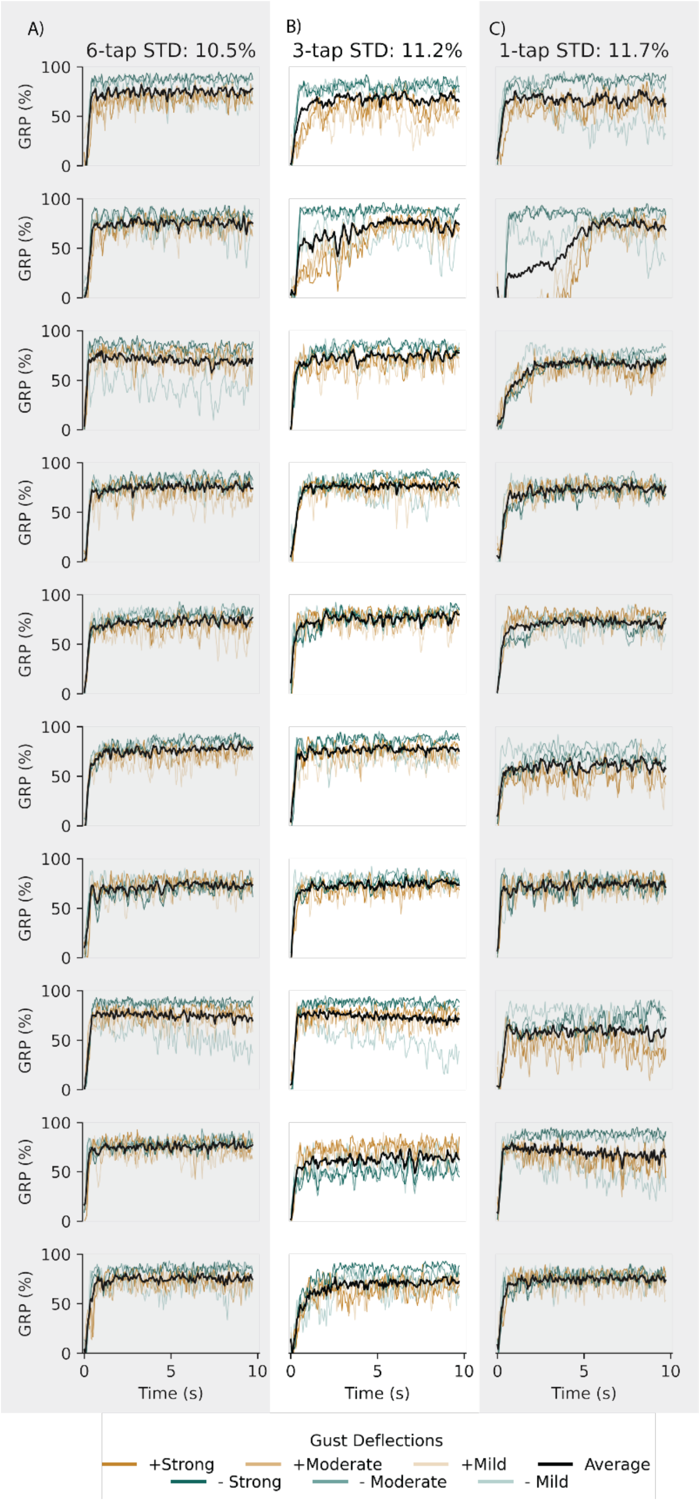


**Supplementary Fig. 6.**

Average gust rejection percentage (GRP) between test iterations (10) at each gust condition (6), for each trained controller (10) at the high-lift flight configuration. Average standard deviation (STD) represents performance consistency between gust conditions for an individual trained controller with (**A**) six, (**B**) three, and (**C**) one pressure tap(s).

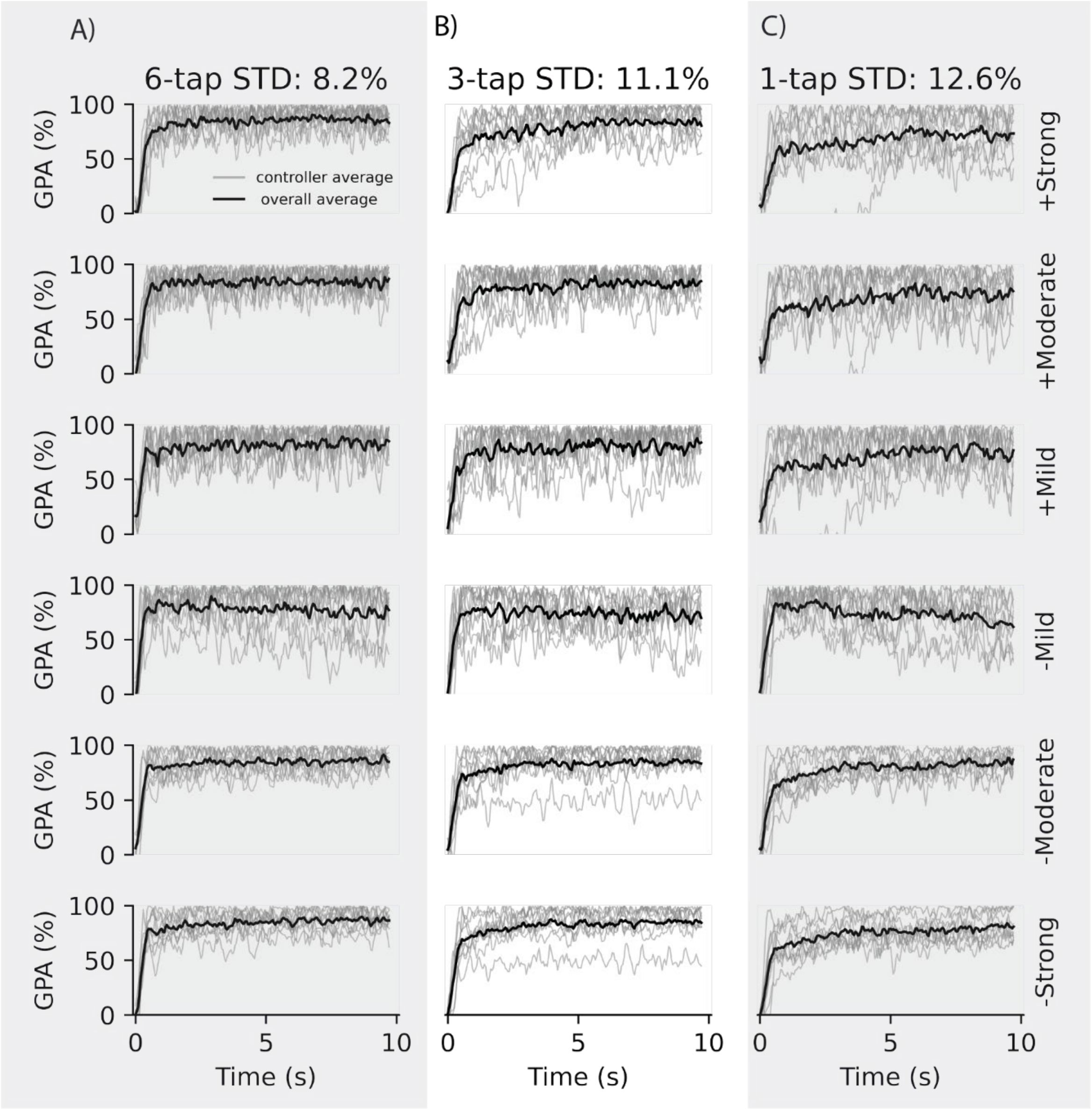


**Supplementary Fig. 7.**

Average gust rejection percentage (GRP) of each trained controller (10) for each gust condition (6) at the high-lift flight configuration. Average standard deviation (STD) represents performance consistency between trained controllers with (**A**) six, (**B**) three, and (**C**) one pressure tap(s), at each gust condition (+Strong, +Moderate, +Mild, -Mild, -Moderate, -Strong).

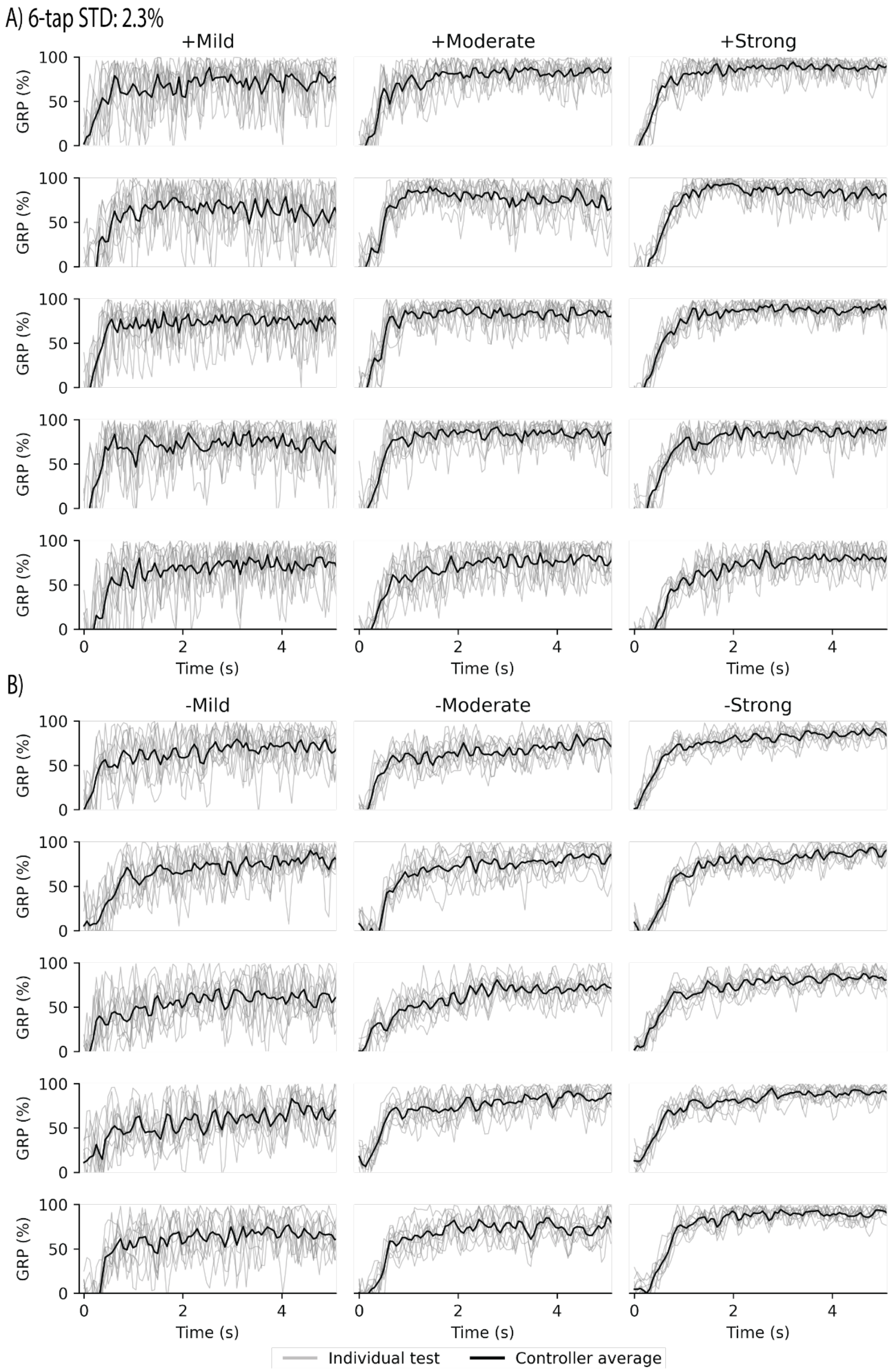
A) 6-tap STD: 2.3%
+Mild
+Moderate
+Strong
GRP (%)
Time (s)
B)
-Mild
-Moderate
-Strong
Individual test
Controller average

C) 3-tap STD: 1.9%

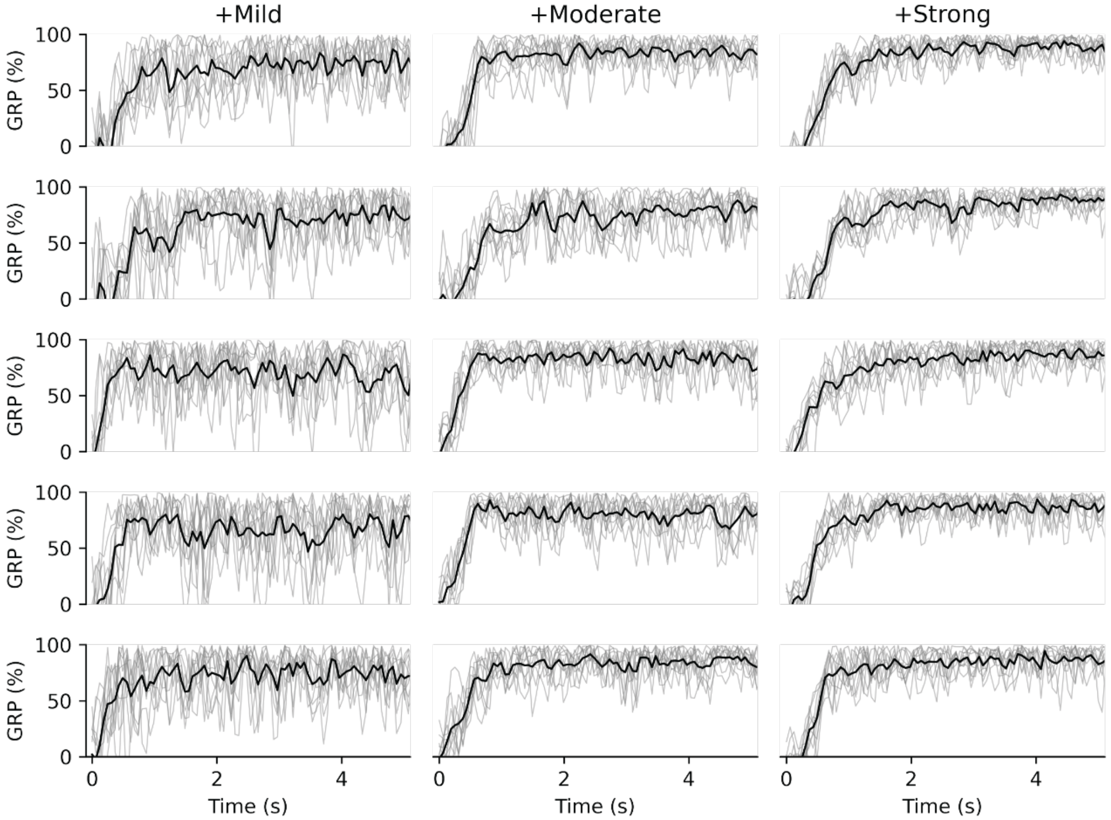


D)

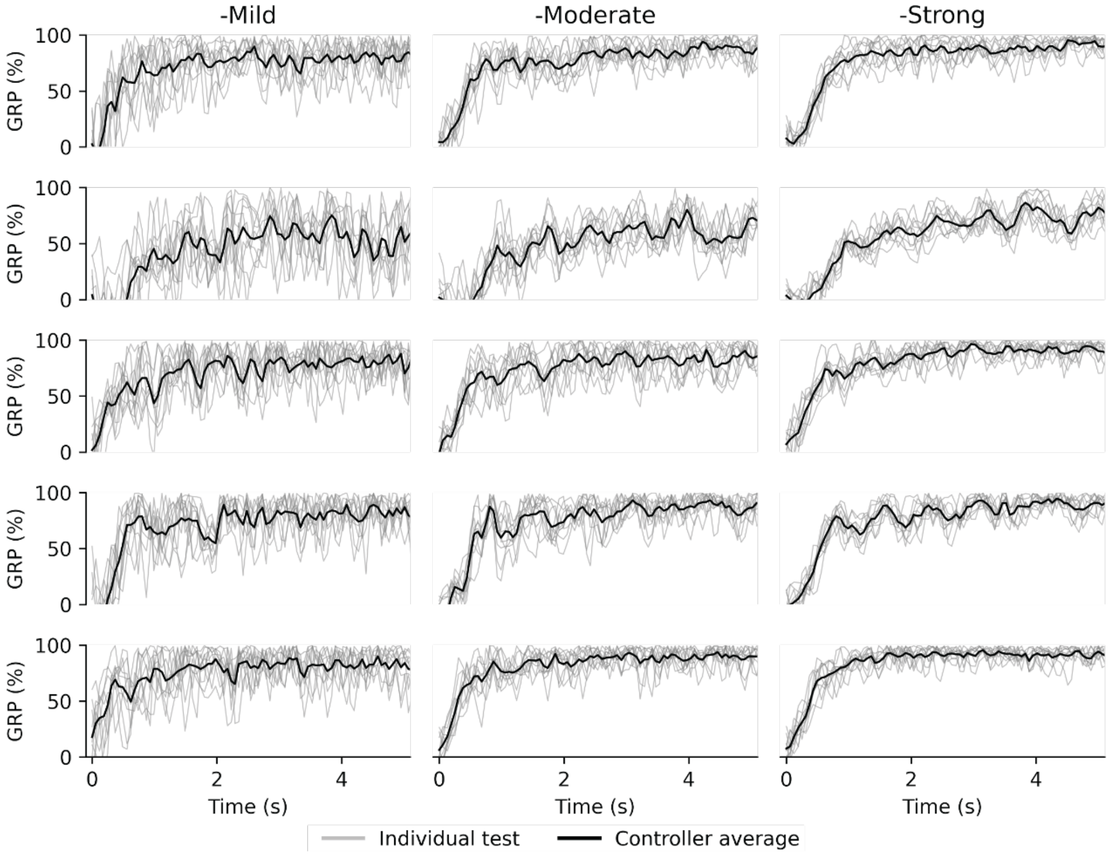

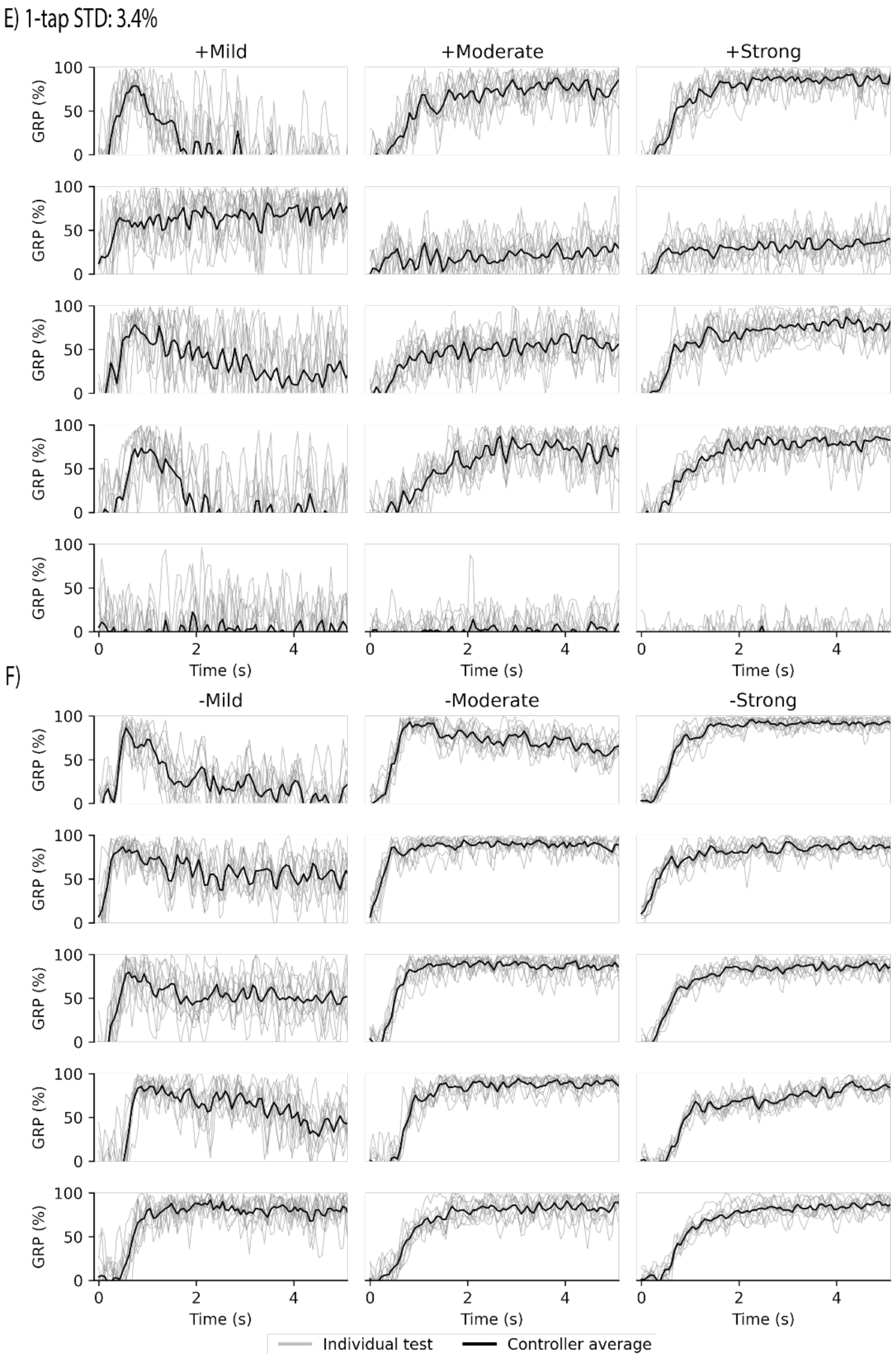


**Supplementary Fig. 8.**

Gust rejection percentage (GRP) of each test iteration (10), during each gust condition (6), for each trained controller (5), using (**A**, **B**) six, (**C**, **D**) three, (**E**, **F**) and one pressure tap(s) for the medium-lift flight configuration. Individual tests (gray) were averaged (black) to provide a general GRP for each trained controller. The average standard deviation (STD) represents performance consistency of individual trained controllers between individual tests for each pressure tap configuration at each gust condition.

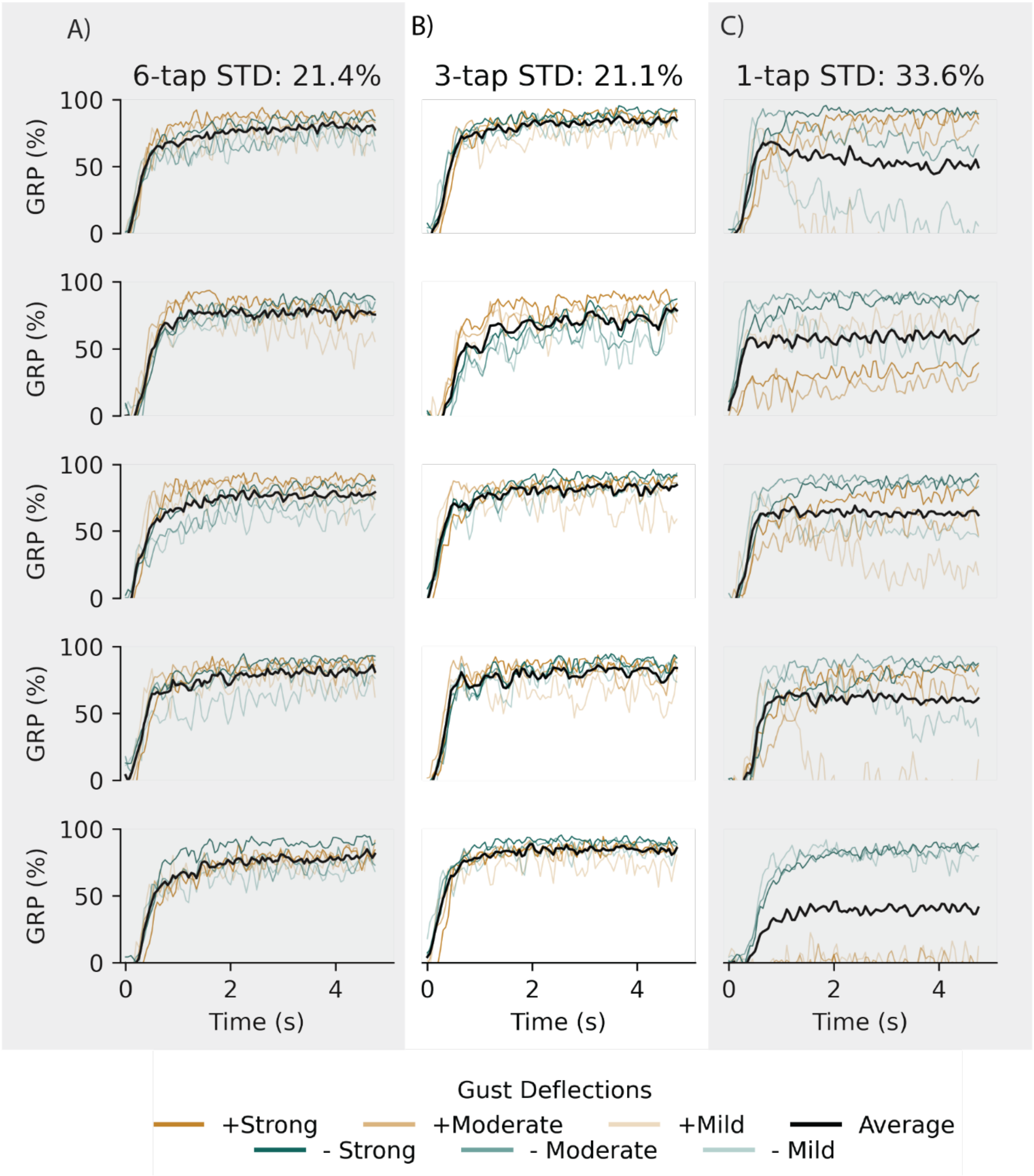


**Supplementary Fig. 9.**

Average gust rejection percentage (GRP) between test iterations (10) at each gust condition (6), for each trained controller (5) at the medium-lift flight configuration. Average standard deviation (STD) represents performance consistency between gust conditions for an individual trained controller with (**A**) six, (**B**) three, and (**C**) one pressure tap(s).

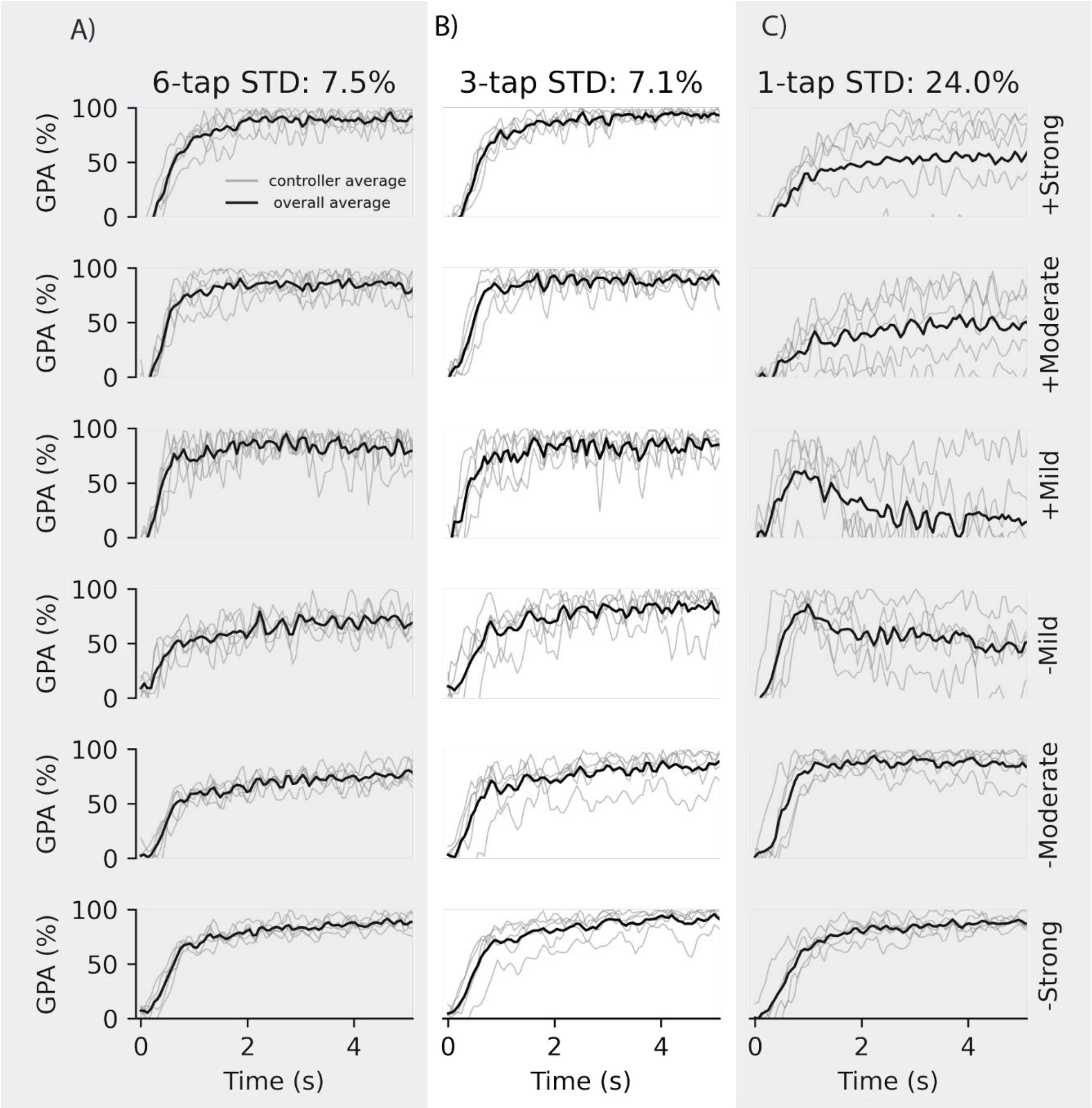


**Supplementary Fig. 10.**

Average gust rejection percentage (GRP) of each trained controller (5) for each gust condition (6) at the medium-lift flight configuration. Average standard deviation (STD) represents performance consistency between trained controllers with (**A**) six, (**B**) three, and (**C**) one pressure tap(s), at each gust condition (+Strong, +Moderate, +Mild, -Mild, -Moderate, -Strong).

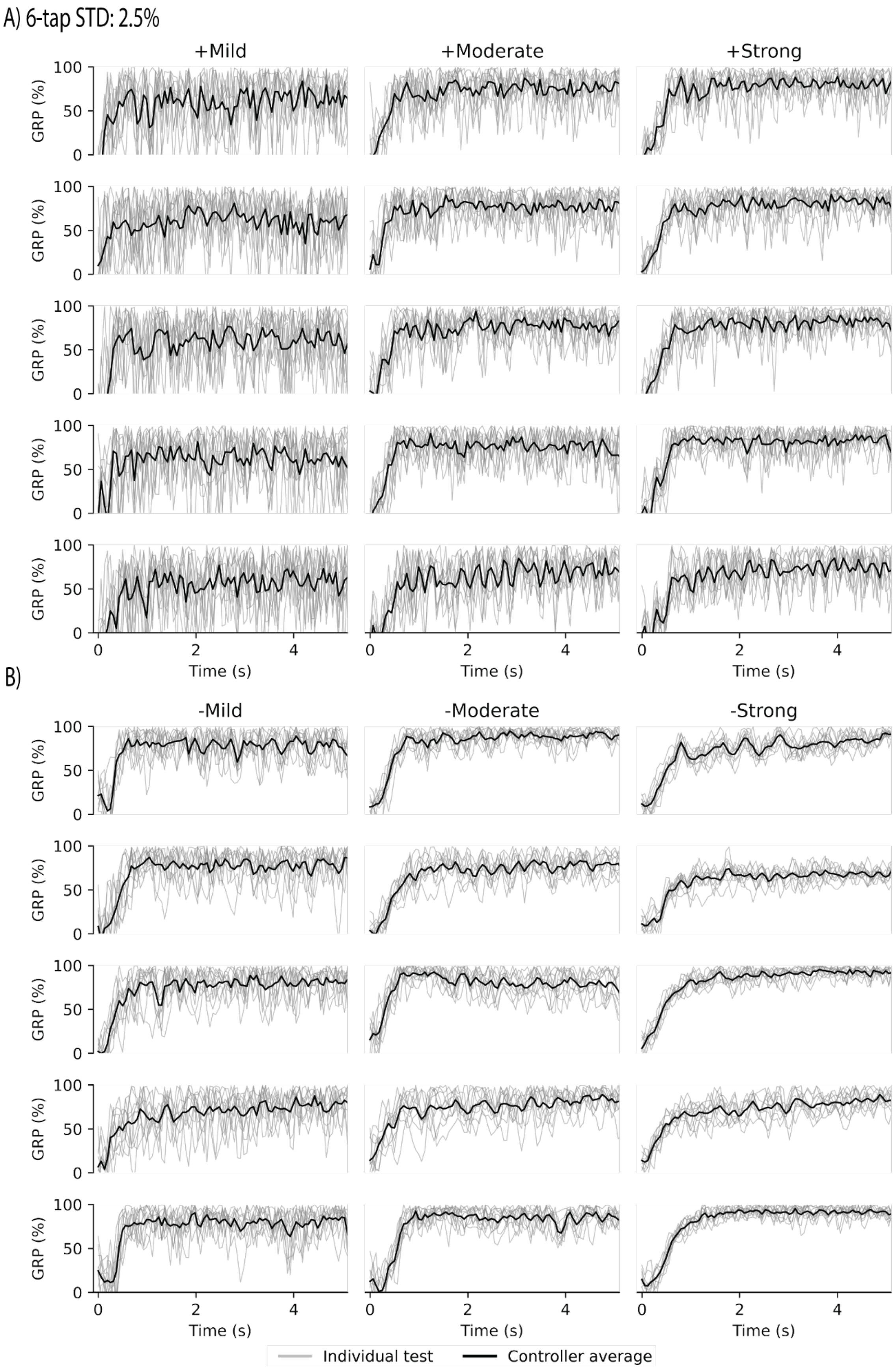
A) 6-tap STD: 2.5%
+Mild
+Moderate
+Strong
GRP (%)
Time (s)
B)
-Mild
-Moderate
-Strong
Individual test
Controller average

C) 3-tap STD: 2.3%

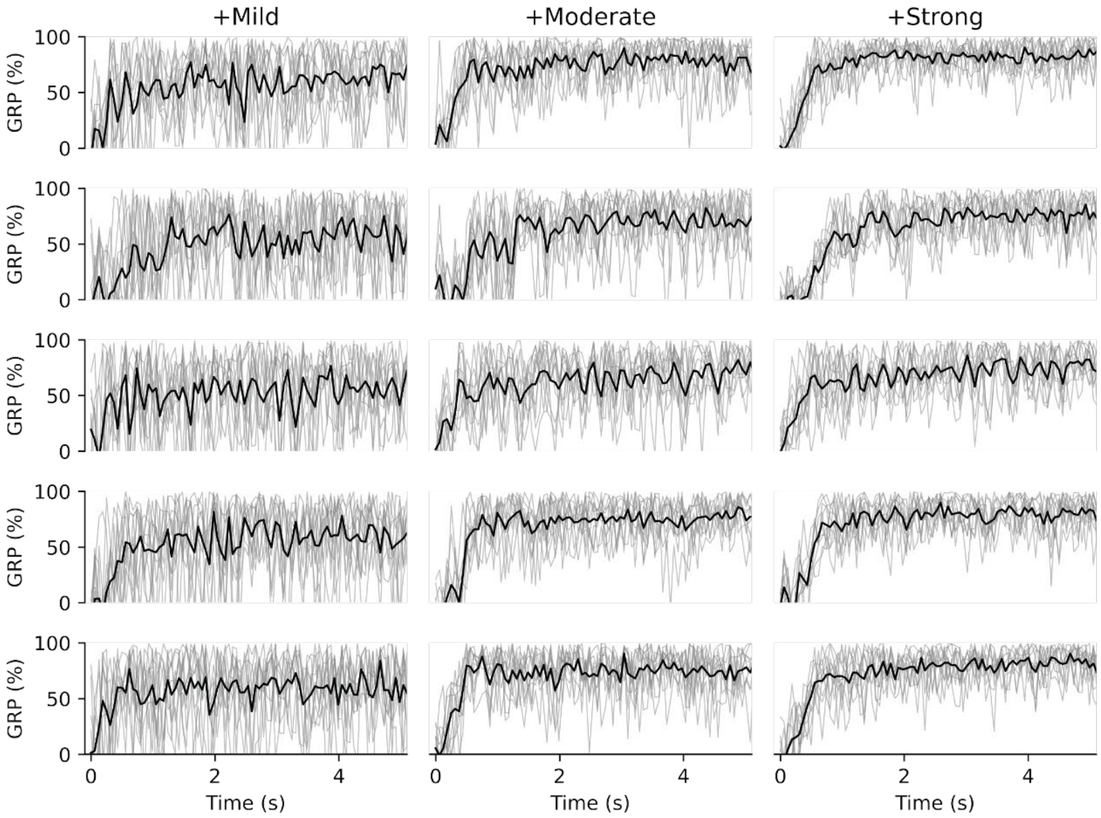


D)

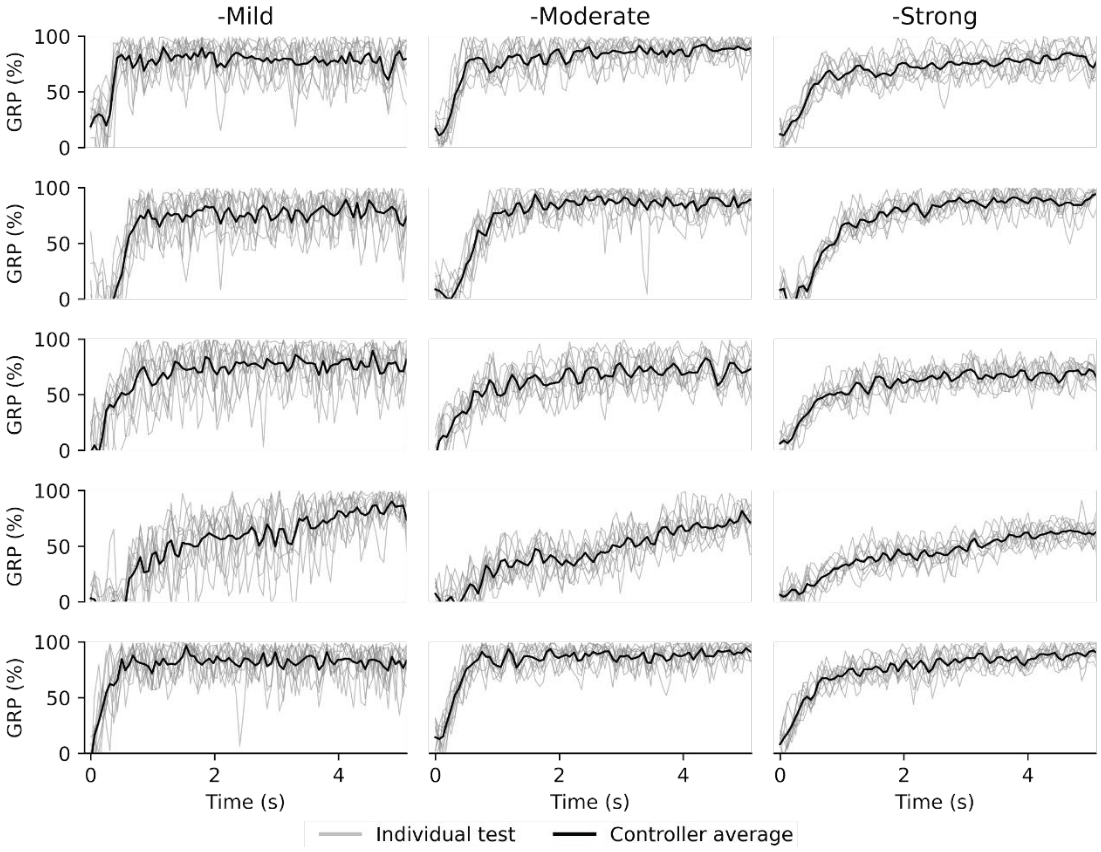

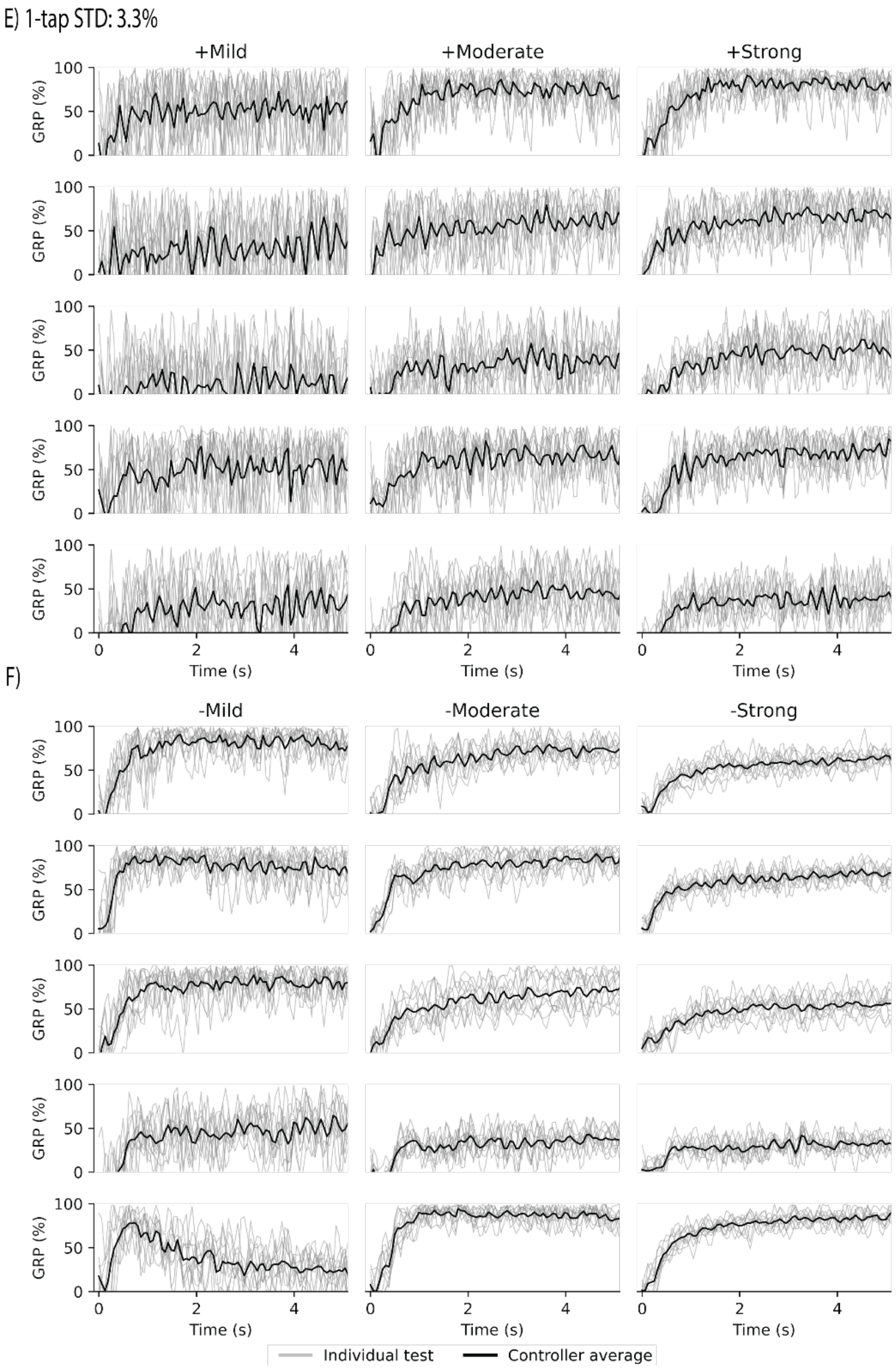


**Supplementary Fig. 11.**

Gust rejection percentage (GRP) of each test iteration (10), during each gust condition (6), for each trained controller (5), using (**A**, **B**) six, (**C**, **D**) three, (**E**, **F**) and one pressure tap(s) for the low-lift flight configuration. Individual tests (gray) were averaged (black) to provide a general GRP for each trained controller. The average standard deviation (STD) represents performance consistency of individual trained controllers between individual tests for each pressure tap configuration at each gust condition.

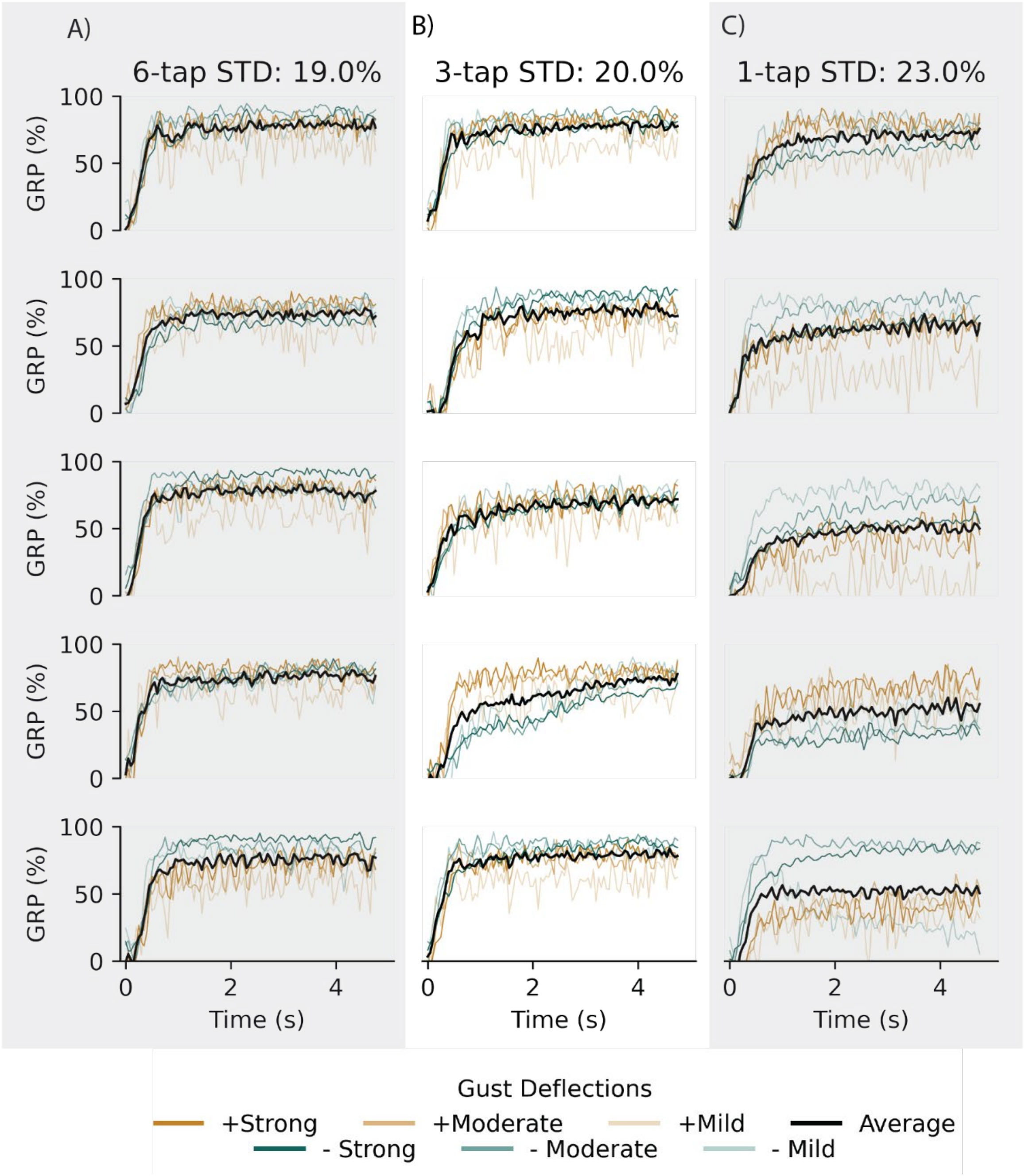


**Supplementary Fig. 12.**

Average gust rejection percentage (GRP) between test iterations (10) at each gust condition (6), for each trained controller (5) at the low-lift flight configuration. Average standard deviation (STD) represents performance consistency between gust conditions for an individual trained controller with (**A**) six, (**B**) three, and (**C**) one pressure tap(s).

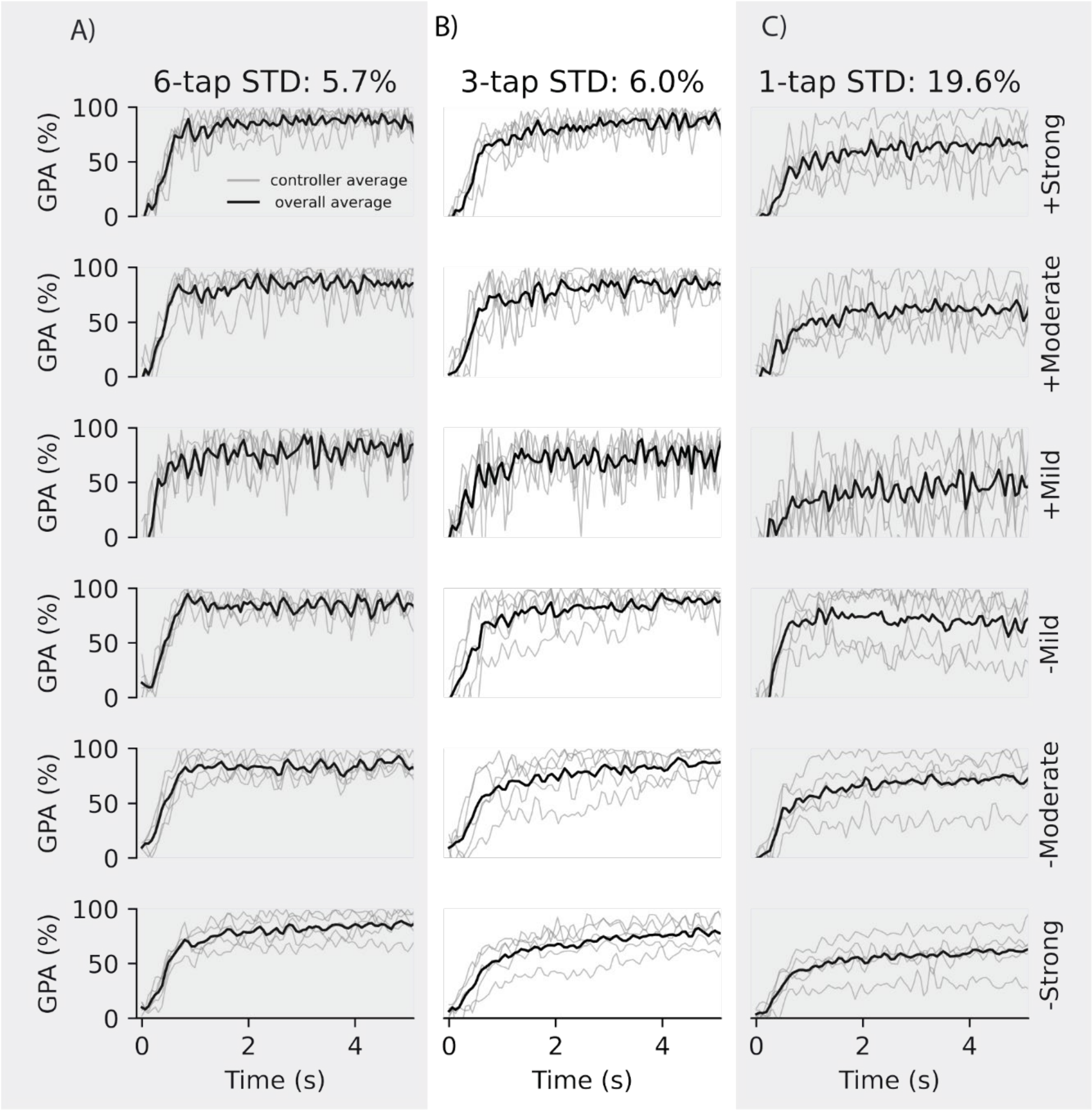


**Supplementary Fig. 13.**

Average gust rejection percentage (GRP) of each trained controller (5) for each gust condition (6) at the low-lift flight configuration. Average standard deviation (STD) represents performance consistency between trained controllers with (**A**) six, (**B**) three, and (**C**) one pressure tap(s), at each gust condition (+Strong, +Moderate, +Mild, -Mild, -Moderate, -Strong).

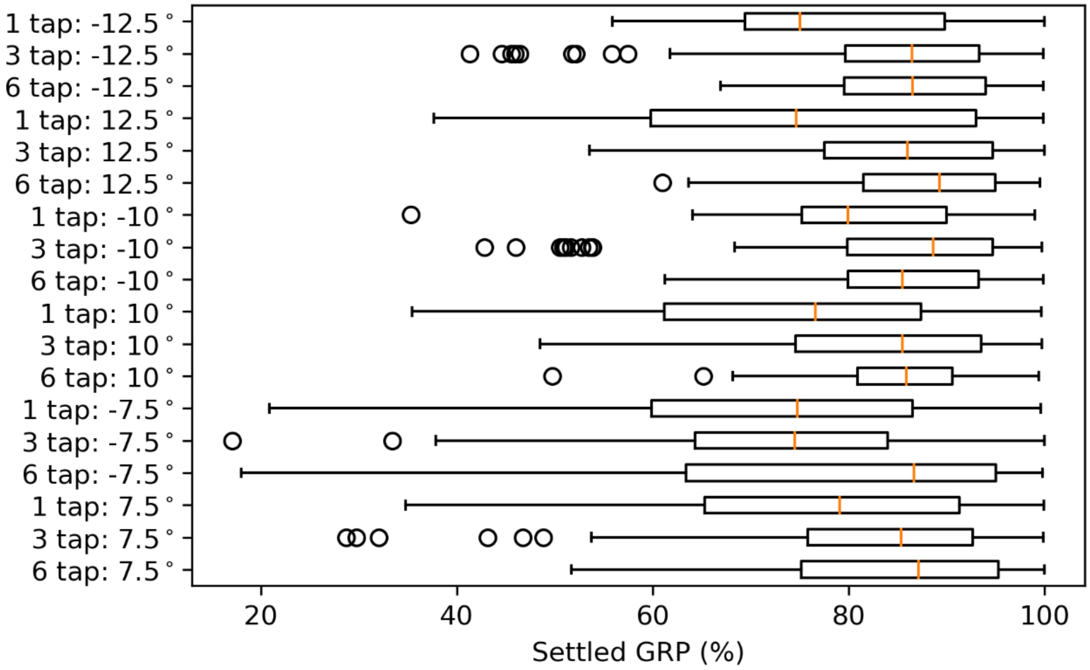


**Supplementary Fig. 14.**

The box-whisker plots for the high-lift flight condition show the distributions (n = 100; center line is the median; box limits are the upper and lower quartiles; whiskers provide the 1.5x interquartile range; circle points represent outliers) of settled gust rejection percentage (GRP) measured for each gust generator deflection (±12.5°, ±10°, ±7.5°) and pressure tap configuration (1, 3, 6). These distributions show the variety of skewness for each testing condition.

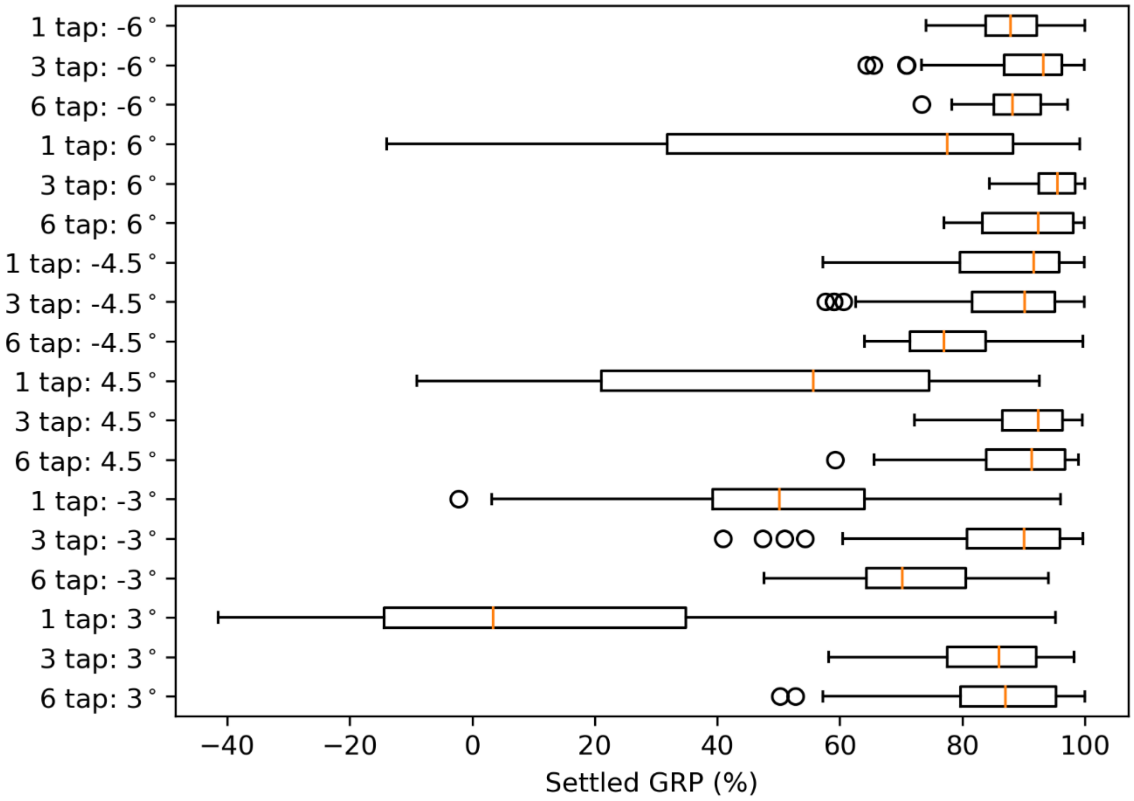


**Supplementary Fig. 15.**

The box-whisker plots for the medium-lift flight condition show the distributions (n = 50; center line is the median; box limits are the upper and lower quartiles; whiskers provide the 1.5x interquartile range; circle points represent outliers) of settled gust rejection percentage (GRP) measured for each gust generator deflection (±6°, ±4.5°, ±3°) and pressure tap configuration (1, 3, 6). These distributions show the variety of skewness for each testing condition.

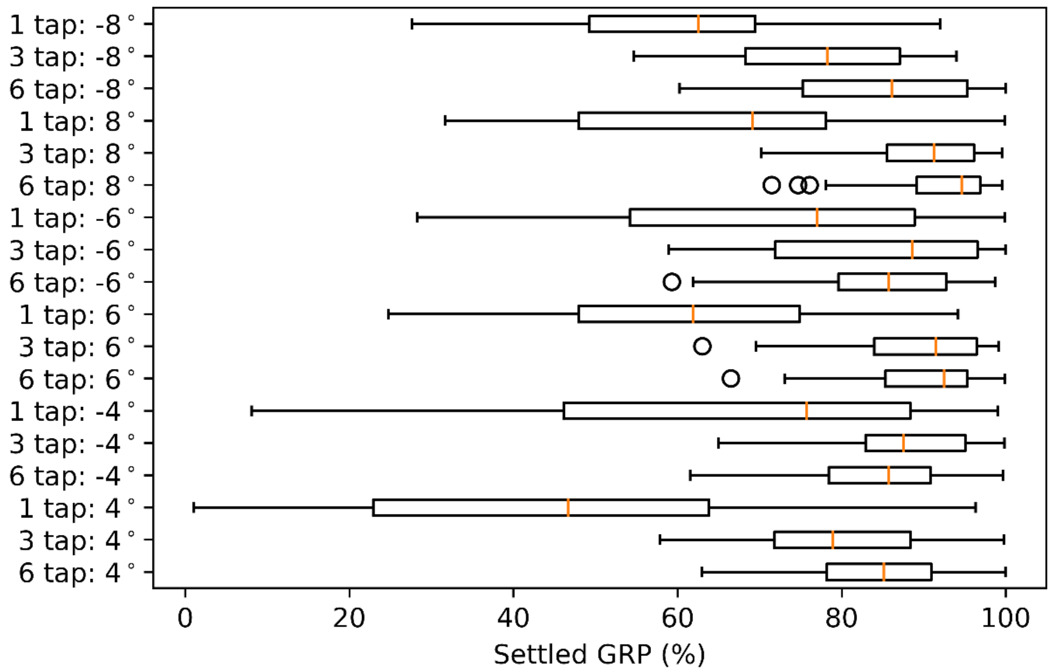


**Supplementary Fig. 16.**

The box-whisker plots for the low-lift flight condition show the distributions (n = 50; center line is the median; box limits are the upper and lower quartiles; whiskers provide the 1.5x interquartile range; circle points represent outliers) of settled gust rejection percentage (GRP) measured for each gust generator deflection (±4°, ±6°, ±8°) and pressure tap configuration (1, 3, 6). These distributions show the variety of skewness for each testing condition.

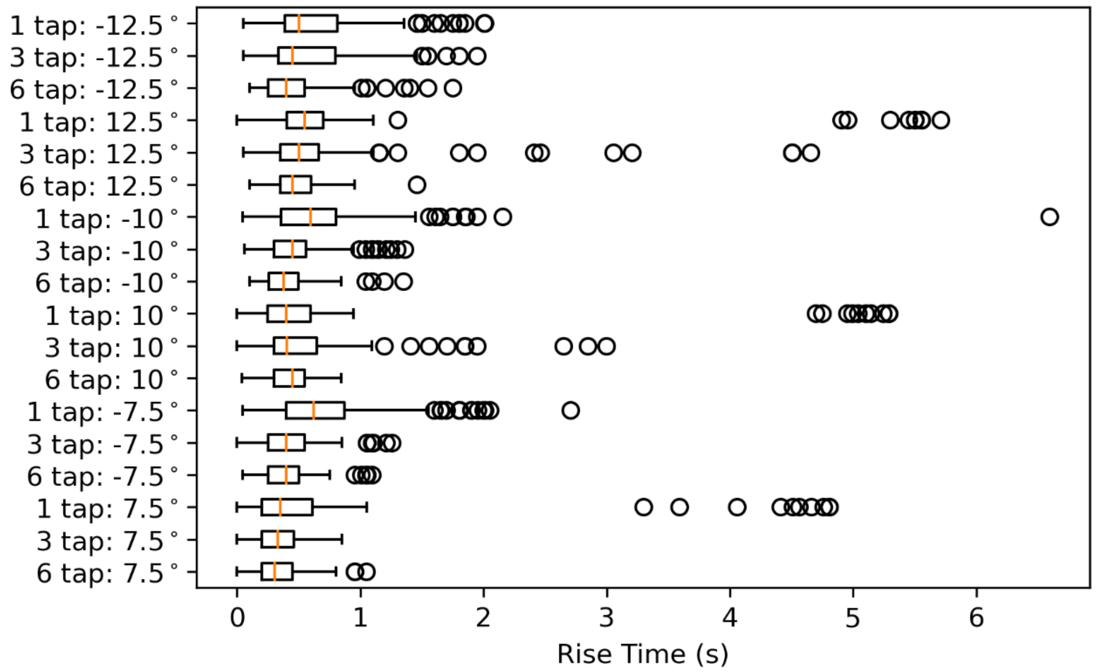


**Supplementary Fig. 17.**

The box-whisker plots for the high-lift flight condition show the distributions (n = 100; center line is the median; box limits are the upper and lower quartiles; whiskers provide the 1.5x interquartile range; circle points represent outliers) of rise time measured for each gust generator deflection (±12.5°, ±10°, ±7.5°) and pressure tap configuration (1, 3, 6). These distributions show the variety of skewness for each testing condition.

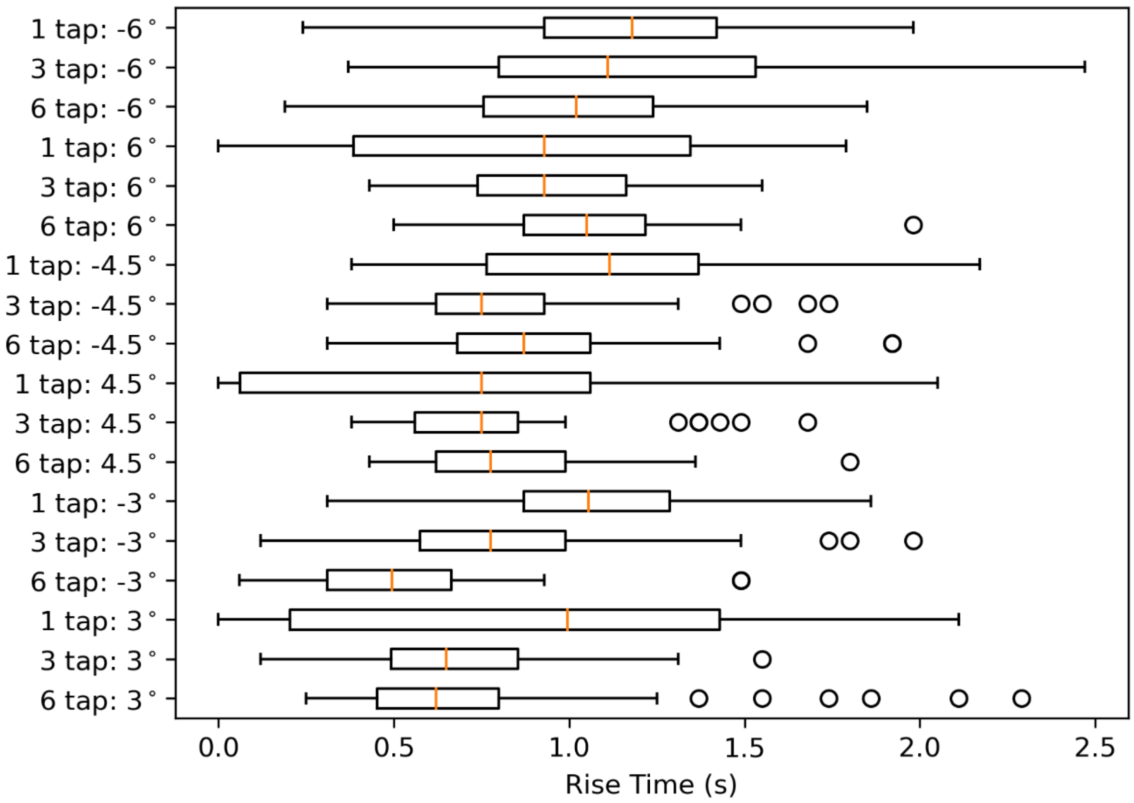


**Supplementary Fig. 18.**

The box-whisker plots for the medium-lift flight condition show the distributions (n = 50; center line is the median; box limits are the upper and lower quartiles; whiskers provide the 1.5x interquartile range; circle points represent outliers) of rise time measured for each gust generator deflection (±6°, ±4.5°, ±3°) and pressure tap configuration (1, 3, 6). These distributions show the variety of skewness for each testing condition.

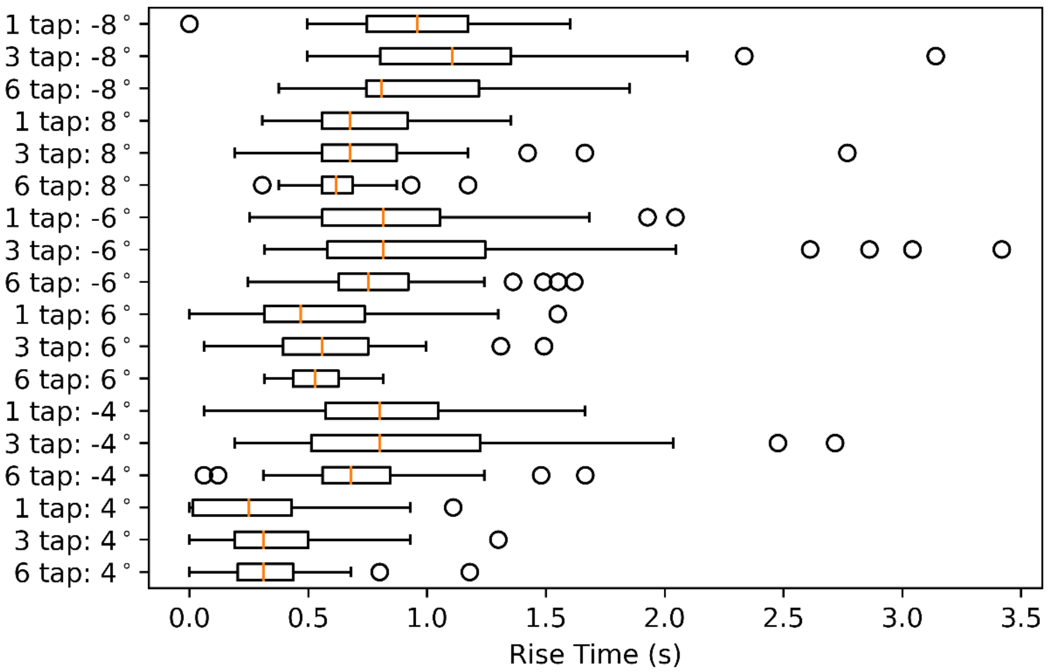


**Supplementary Fig. 19.**

The box-whisker plots for the low-lift flight condition show the distributions (n = 50; center line is the median; box limits are the upper and lower quartiles; whiskers provide the 1.5x interquartile range; circle points represent outliers) of rise time measured for each gust generator deflection (±4°, ±6°, ±8°) and pressure tap configuration (1, 3, 6). These distributions show the variety of skewness for each testing condition.

| Flight Condition | High-lift | Med-lift | Low-lift |
|---|---|---|---|
| $L_B$ (N) | 3.5 | 2.2 | 1.2 |
| $U$(m s$^{-1}$) | 10 | 15 | 10 |
| α(°) | 10±1 | 4±1 | 4±1 |
| Training Deflection (°) | ±[3.5 : 13.5] | ±[0.5 : 7.5] | ±[1 : 9] |
| Testing Deflection (°) | ±[7.5, 10, 12.5] | ±[3, 4.5, 6] | ±[4, 6, 8] |
| $\Delta L_B$ (N) | [-.17 , -.15, -.08, .09, .10, .14] | [-.61, -.43, -.26, 0.21, .36, .51] | [-.35, -.24, -.14, .13, .22, .28] |
| Gust Duration (s) | 10 | 5 | 5 |
| Action Space (ΔV) | ±[0, 0.1 , 0.2, 0.6] | [-0.25, 0, 0.25] | [-0.25, 0, 0.25] |
| Pressure Signal States | | [-2.5 : 2.5] | |
| MFC Signal States | | [-1 : 1] | |

**Supplementary Table 1.**

Environmental training and testing considerations for each flight condition (High-lift, Med-lift, Low-lift). Features that changed between flight conditions included the baseline lift, $L_B$, the velocity, $U$, the angle of attack, α, the gust generator deflections used during training and testing, the generated change in lift for tested gust deflections, $\Delta L_B$, the duration of each tested gust, and the changes in MFC voltage signal that created the policy action spaces. The overall policy state spaces, containing the pressure signals and macro-fiber composite (MFC) voltage signals, remained constant between flight conditions.

| Parameter | Value |
|---|---|
| Episodes | 1000 |
| Steps per Episode | 200 |
| Batch Size | 5 |
| Epochs | 4 |
| Timestep Update | 20 |
| Clipping Factor (ϵ) | 0.2 |
| Discount Factor (γ) | 0.99 |
| Smoothing Factor (λ) | 0.95 |
| Learning Rate | $3\times10^{-5}$ |

**Supplementary Table 2.**

Hyperparameters used during proximal policy optimization controller training. Due to challenges associated with training in hardware environments, many hyperparameters were determined from previous work in a similar macro-fiber composite (MFC) camber morphing wing environment[52]. One exception was the learning rate, which was tuned manually through a series of preliminary training sessions.
